# From self-assembly to controlled release: synthesis and properties of dynamic imine-based Gemini surfactants for curcumin delivery

**DOI:** 10.1016/j.fochx.2026.103635

**Published:** 2026-02-08

**Authors:** Wenbo Zhao, Heng Zhang, Wenwen Yu, Fengbo Zhu, Jianjun Xu, Quanxin Xu, Hongwei He, Fuyong Liu, Qiang Zheng

**Affiliations:** a College of Materials Science and Engineering, Taiyuan University of Technology, Taiyuan 030024, China; b R&D Center, XiYueFa International Environmental Protection New Material Co., Ltd, Taiyuan 030006, China; c Department of Polymer Science & Engineering, Zhejiang University, Hangzhou 310027, China

**Keywords:** Gemini surfactants, pH-responsiveness, self-assembly, surface activity, controllable release of curcumin

## Abstract

Surfactants based on dynamic covalent bonds have attracted great interest in the past few decades due to the coexistence of covalent and non-covalent bonds. As a novel structure of surfactants, Gemini surfactants exhibit excellent surface activity and aggregation behavior. This work synthesized dicot quaternary ammonium salts containing aldehyde precursors, which can self-bind with primary amines of different chain lengths in water to undergo Schiff base reactions and generate pH responsive dynamic surfactants. Different linking groups and hydrophobic chain lengths were used to synthesize Gemini surfactants. The micelle aggregation behavior and surface activity of Gemini surfactants were systematically studied through surface tension, fluorescent probes, and conductivity. Particularly, the Gemini surfactant with 6 carbon atoms between amphiphilic groups and 10 carbon atoms in hydrophobic tail chain change the surface tension of water to 28.88mN·m^−1^ with a critical micelle concentration of 0.117 mmol·L^−1^.Products with different chain lengths and linking groups exhibit different micelle aggregation characteristics. Dynamic light scattering, rheology, and cryogenic transmission electron microscopy were used to study their high concentration aggregation behavior and the self-assembled vesicles and worm-like micelles of Gemini surfactants were revealed finally. The ability of micelles to encapsulate and release curcumin was studied by UV-visible spectroscopy and fluorescence spectroscopy, and it was found that they have excellent encapsulation ability for curcumin. Different from micelles, the aggregation behavior of Gemini surfactants changed with the concentration of them in aqueous solution increased.

## Highlights

• Synthesis of a series of novel pH-responsive Gemini surfactants with tunable imine linkers and chain lengths.
• The surfactants exhibit excellent surface-active properties.
• The self-assembly of Gemini surfactants in aqueous solution was discovered through rheological and Cryo-TEM.
• The pH-responsiveness of the Gemini surfactants was demonstrated and applied to control the release of curcumin.

## 1 Introduction

Surfactants are amphiphilic molecules featuring distinct polar (hydrophilic) heads and nonpolar (hydrophobic) tails and the dual structure governs their fundamental behaviors in solution (Sanchez-Huerta, Zhang, Alahmari, Humam, & Hong, 2025; Yi et al., 2026). At low concentrations, they adsorb at interfaces, exhibiting surface activity; above a critical concentration, they self-assemble into various aggregates such as micelles, vesicles, and liquid crystalline phases (Le Bastart et al., 2025; Lu, Liu, Dong, & Li, 2025; Varytimiadou & Giesselmann, 2025). Different structures of surfactants lead to a rich array of aggregates, including spherical micelles, rod-like micelles, wormlike micelles, vesicles, coacervates, gels, liquid crystal, and so on, which subsequently influence their solubilization efficiency, rheological properties, flow characteristics, spreading behavior, and encapsulation efficacy (Ghosh, Seth, & Purkayastha, 2018; Pinthong et al., 2025; Qiao, Fan, Ding, & Fang, 2021; Turk, Kogej, & Hansson, 2025; Wang et al., 2025). Among diverse surfactant classes, Gemini surfactants, which consist of two identical amphiphilic moieties linked by a spacer, have garnered significant interest due to their superior surface activity, enhanced solubilization power, and tunable molecular geometry (Liu & Yu, 2025; Prajapati et al., 2025; Zhao & Wang, 2017). These attributes make them particularly promising for advanced applications, including drug and gene delivery (Peng et al., 2021; Rajput et al., 2020; Zhang et al., 2018).

However, conventional Gemini surfactants are typically static systems; their properties and assembled structures are fixed upon synthesis, lacking responsiveness to environmental cues. This limits their utility in emerging “smart” applications such as controlled drug release or adaptive catalysis, where dynamic, stimulus-responsive behavior is essential (Hu et al., 2025; Li et al., 2025). In this context, the integration of dynamic covalent chemistry into surfactant design offers a powerful strategy to create adaptive molecular systems (Kim et al., 2025; Liu, Wang, & Wang, 2025; Yan, 2025). Dynamic covalent bonds, such as disulfide bonds (Sokjorhor, Phantan, Ratanathawornkit, & Crespy, 2025), boronic esters (Guo et al., 2024), and imine bonds, can undergo reversible cleavage and reformation under specific conditions, endowing materials with adaptivity, self-healing, and stimuli-responsiveness. The imine bond (Schiff base), in particular, is highly attractive due to its facile synthesis, mild formation conditions, and high sensitivity to pH changes, making it an ideal candidate for designing pH-responsive materials (Fan et al., 2024; Yang, Park, Kim, & Kim, 2024). While imine chemistry has been employed to confer stimulus-responsiveness to single-chain surfactants (Li & Liu, 2022; Lu, He, Zhou, & Zhang, 2021), its strategic incorporation into Gemini architectures—particularly at the critical junction between the hydrophilic headgroup and hydrophobic tail—remains underexplored. Such a design could allow precise and reversible control over self-assembly: cleavage of the imine bond would convert the Gemini surfactant into two single-chain precursors, dramatically altering its aggregation behavior and functional properties in response to pH.

Curcumin, a natural polyphenol derived from turmeric rhizome, possesses a broad spectrum of biological activities, including anti-inflammatory, antioxidant, anti-tumor, and neuroprotective effects (Nisoa et al., 2025; Rai, Feitosa, Ingle, & Golinska, 2025). These properties hold considerable promise for its application in pharmaceuticals and functional foods. However, its therapeutic potential is severely hampered by intrinsic drawbacks such as extremely poor aqueous solubility, low chemical stability, rapid in vivo metabolism, and consequently, low oral bioavailability. To address these challenges, various nanocarrier systems have been explored to enhance the solubility, stability, and targeted delivery of curcumin. Among them, surfactant-based micelles serve as a common and effective nano-delivery platform (Kuang et al., 2020; Zhao, Jia, Zhang, Wang, & Zhang, 2024). Through hydrophobic interactions, they can encapsulate curcumin within their core, thereby significantly improving its aqueous dispersibility, stability, and systemic circulation time. Moreover, micellar systems can be functionally designed to respond to specific environmental stimuli (e.g., pH, temperature, or enzymes), enabling controlled drug release at the target site (Fidalgo, Costa, Garcia-Rìo, & Gerola, 2025). This is particularly advantageous for delivering curcumin to pathological sites like tumors or inflamed tissues, which often exhibit a weakly acidic microenvironment. A pH-responsive micelle system can facilitate the controlled release of curcumin under such acidic conditions, thereby enhancing its local efficacy while minimizing systemic toxicity. Therefore, the development of an intelligent, stimulus-responsive micellar delivery system is of significant importance for advancing the practical biomedical application of curcumin.

Herein, we designed and synthesized a novel pH-responsive Gemini surfactant featuring a dynamic imine bond precisely at the hydrophilic-hydrophobic interface. We hypothesize that this structure will not only exhibit excellent surface activity but also undergo reversible assembly-disassembly in response to pHs. This work aims to: (i) characterize its basic properties, including *cmc*, and demonstrate its pH-responsive behavior; (ii) investigate its concentration-dependent self-assembly, with a focus on the formation of large aggregates and viscoelastic wormlike micelles at high concentrations and (iii) showcase its application as an intelligent delivery vehicle using curcumin as a model bioactive compound. We hope this work not only to present a high-performance carrier for curcumin but also to provide a general strategy based on dynamic covalent chemistry for the design of the next generation of intelligent soft materials.

## 2 Experimental

### 2.1 Materials

4-hydroxybenzaldehyde(>99%), 1,2-dibromoethane(>99%), *N*_*1*_*,N*_*1*_*,N*_*3*_*,N*_*3*_-tetramethylpropane-1,3-diamine(>99%), *N,N,N',N'*-tetramethyl-1,6-hexanediamine(>99%), 1-hexamine(>99%), 1-octylamine(>99%), 1-decamine(>99%), curcumin(>98%), tris (hydroxymethyl) aminomethane hydrochloride(>99%), acetic acid(>99.5%), sodium acetate anhydrous(>99%), sodium hydroxide(>99%), hydrochloric acid(36%), pyrene(>99.9%), methanol(>99.9%) and all the other organic solvents used in the research were analytical-grade products from Admas-beta. Water was distilled by a quartz water purification system (R = 18 MΩ·cm).

### 2.2 Synthesis of Gemini surfactants

**Scheme 1:**
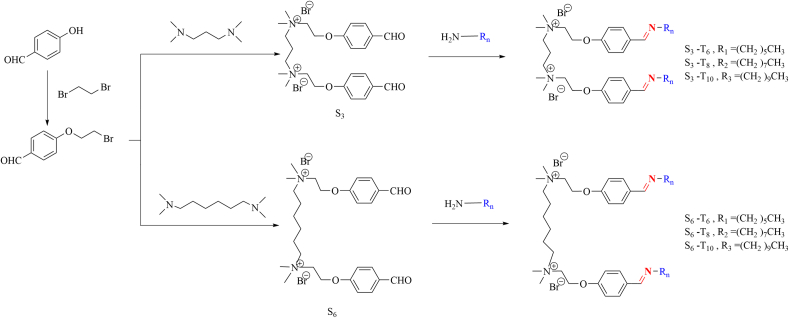
Synthesized route of Gemini Surfactants.

The synthesized route of Gemini Surfactants was exhibited in Scheme 1.

#### 2.2.1 Synthesis of 4-(2-bromoethoxy) benzaldehyde

In a 500 mL round-bottom flask, 1,2-dibromoethane (35 mL), acetonitrile (150 mL) and K_2_CO_3_ (22.6 g) were mixed and refluxed by heating. 4-Hydroxybenzaldehyde (10.0 g) was dissolved in acetonitrile (100 mL) and then the solution was dropwise added. The mixture was continuously stirred for 24 h under reflux and progress was controlled by TLC. After cooling to room temperature, the mixture was extracted using rotary evaporator and the unreacted solvent is evaporated by it at 60 °C. The crude product was further purified by column chromatography (eluent: ethyl acetate/petroleum ether = 1:4 v/v) to yield white crystals (12.1 g). Target product structure was characterized by ^1^H NMR and ^13^C NMR (Fig.S1, Supplementary material).

#### 2.2.2 Synthesis of S_3_ and S_6_

In a 250 mL round-bottom flask, 4-(2-bromoethoxy) benzaldehyde, (12.6 g, 0.055 mol) and *N*_*1*_*, N*_*1*_*, N*_*3*_*, N*_*3*_-tetramethylpropane-1,3-diamine (4.0 mL, 0.024 mol) were mixed in acetonitrile (150 mL) and refluxed for 24 h under a nitrogen atmosphere. After cooling to room temperature, the solvent was removed under reduced pressure and then the crude product was washed with n-hexane to yield 1,3-Bis (N, N-dimethyl-N-p-oxybenzylethylammonium bromide) propane (S_3_) as a white solid (6.7g).

Similarly, 1,6-Bis (N, N-dimethyl-N-p-oxybenzylethylammonium bromide) hexamine (S_6_) as a white solid (12.3g) was synthesized by reacting of 4-(2-bromoethoxy) benzaldehyde (12.6 g, 0.055 mol) and *N*, *N*, *N*', *N*'-tetramethyl-1,6-hexanediamine (5.2 mL, 0.024 mol) in acetonitrile (150 mL). Notably, the reaction time was reduced (12 h reflux) and the yield was increase due to alkyl chain spacer between two quaternary ammonium salt heads was extended.

The molecular structure was characterized by ^1^H NMR and ^13^C NMR (BRUKER AVANCE 400 MHz spectrometer, Bruker, Germany), (Fig.S2, Supplementary material) and HRMS (Thermo Scientific Q Exactive, USA), (Fig.S3, Supplementary material).

Yield of S_3_: 57%. ^1^H NMR (400 MHz, D_2_O, ppm) δ: 9.52 (s, 2H), 7.59 (d, *J* = 8.8 Hz, 4H), 6.94 (d, *J* = 8.7 Hz, 4H), 4.54 (s, 4H), 3.94 (s, 4H), 3.61 – 3.52 (m, 4H), 3.28 (s, 12H), 2.48 (s, 2H). ^13^C NMR (D_2_O, ppm) δ: 17.35 (CH_2_, spacer), 52.32 (NCH_3_), 60-63(NCH_2_), 63.52(CH_2_, alkyl), 115-165(C, phenyl), 194.45(C, carbonyl). MS of S_3_: Calcd:507.0(M-Br^-^). Found: m/z=507.2.

Yield of S_6_: 98%. ^1^H NMR (400 MHz, D_2_O, ppm) δ 9.64 (s, 2H), 7.78 (d, *J* = 8.8 Hz, 4H), 7.03 (d, *J* = 8.8 Hz, 4H), 4.52 (s, 4H), 3.79 (s, 4H), 3.43 – 3.33 (m, 4H), 3.15 (s, 12H), 1.79 (s, 4H), 1.38 (s, 4H). ^13^C NMR (D_2_O, ppm) δ: 22-26 (CH_2_, spacer), 52.04 (NCH_3_), 62.26 (NCH_2_), 65.28 (CH_2_, alkyl), 115-165(C, phenyl), 194.44(C, carbonyl). MS of S_3_: Calcd:549.0(M-Br^-^). Found: m/z=549.2.

FT-IR spectra were obtained on an INVENIO-S instrument (USA) with wave numbers ranging from 500–4000 cm^−1^.

#### 2.2.3 Preparation of the Gemini surfactants solution

The Gemini surfactants were formed at 25°C through dynamic covalent chemistry by preparing a stoichiometric aqueous mixture (1:2 molar ratio) of aldehyde-functionalized precursors (S_3_ or S_6_) and multifunctional amine linkers.

### 2.3 Measurement of surfactants performance

#### 2.3.1 Surface tension

The surface tension (*γ*) was measured with a Krüss DSA25 tensiometer at 25.0 ± 0.1°C. Instrument calibration was performed prior to sample analysis with distilled water (18.2 MΩ·cm^-1^, reference value 71.93 mN·m^-1^, Fig.S4, Supplementary material). Successive measurements were recorded at 3-minute intervals until consecutive γ values exhibited <0.03 mN·m^-1^ variation over three measurement cycles to ensure adsorption-desorption equilibrium at the air/water interface.

#### 2.3.2 Fluorescence experiment

Fluorescence emission spectra of Nile red (NR) were measured on a Hitachi F-7100 spectrofluorometer at 25°C. The excitation wavelength was set at 550 nm and the excitation and emission slit widths were both set at 5 nm. The scanning rate was set to 240 nm·min^-1^ within the range of 560∼800 nm. The samples were prepared by injecting 10.0μL of 2.00 mM NR solution in anhydrous tetrahydrofuran (THF, ≥99.9%) into 5.00 mL surfactant solution. Molecular dispersion equilibration was ensured by maintaining samples in a thermostatic bath (25.0 ± 0.1°C) for ≥12 h prior to measurement.

#### 2.3.3 Conductometric experiment

Electrical conductivity measurements were performed using a digital conductivity meter (DDS-11A) equipped with a platinum electrode. Thermostatic control (accuracy ± 0.05°C) was maintained, ensuring <0.02°C spatial variation within the measurement cell. Every sample was measured for three times, and their average value was taken as the conductivity test result. Before measurement, the instrument was calibrated through three-point calibration using standards (84μS/cm^-1^, 1413μS/cm^-1^, 12.88mS/cm^-1^) and adjusting the constant to 1.13.

### 2.4 Dynamic light scattering

Hydrodynamic size distributions were characterized by dynamic light scattering using a Malvern Zetasizer Nano SZ-100 system. The samples were filtered through a 0.45 μm syringe filter before the measurements and allowed to equilibrate inside the DLS optical system chamber for 5 min before the beginning of the measurement. Three runs were performed with automated measurement and attenuation procedures and a 60 s equilibration time using water as a dispersant. The average value of three measurements was taken as the final value for the Z-Average. Colloidal stability was ensured by keeping the temperature at 25.0 ± 0.1°C for 24 h before experiment.

### 2.5 Rheological experiment

Rheological characterization was conducted using a TA Instruments AR-G2 rotational rheometer equipped with a precision-machined titanium cone-and-plate geometry (25 mm diameter, 0.054 rad cone angle). The sample of surfactant solution were placed between cone plate and base plate and all measurements were performed in the linear viscoelastic region.

### 2.6 Cryo-transmission electron microscopy (Cryo-TEM)

Cryo-TEM images of the samples were obtained by using a FEI Talos F200C TEM instrument running at 200 kV of acceleration. A volume of ∼5 μL surfactant solution was first placed on a copper grid, and the grid was blotted with a piece of filter paper to form a thin solution film. The grid was quenched rapidly with liquid ethane at −180 °C and then transferred into liquid nitrogen (−196 °C) for storage until observation.

### 2.7 pH responsiveness and self-assembly structure of surfactant-curcumin systems

#### 2.7.1 UV–visible spectroscopic

The gathering state of surfactant-curcumin systems was studied using a Lambda 1050+ UV-vis spectrometer in the wavelength range of 350-700 nm. Gemini surfactant solutions were prepared and sonicated in an aqueous medium at 25°C for 30 minutes. 10μL ethanol solution of curcumin (1mg·L^-1^) was added to an 10mL aqueous solution of S_3_-series or S_6_-series Gemini surfactant (3 times *cmc*) at different pHs. It was allowed to stand for 2 hours to facilitate the full binding of curcumin and micelles after stirring. The acidic environment was obtained by dissolving the surfactants in a acetic acid-sodium acetate solution at pH=5 and the alkaline environment was obtained by dissolving the surfactants in a Tris-HCl solution at pH=8. The UV-vis spectra of curcumin at different pH values were recorded.

#### 2.7.2 Fluorescence spectroscopic studies

All fluorescence measurements were conducted on a Hitachi F-7100 spectrofluorometer. The emission spectra of the Gemini surfactant-curcumin systems were measured at excitation wavelength of 430 nm and a scan rate of 240 nm·min^-1^ within the range of 450∼800 nm. Both the excitation and emission slit width were kept at 5 nm.

Emission spectra of pyrene were noted by adding Gemini surfactant-curcumin systems to a fixed pyrene concentration (0.2 μM).The fluorescence experiments of Gemini surfactant-curcumin systems with pyrene probe were measured at excitation wavelength of 335 nm and a scan rate of 240 nm·min^-1^ within the range of 350∼500 nm. The excitation slit width was kept at 5 nm, while the emission slit width was at 2.5 nm.

## 3 Results and discussion

### 3.1 Formation of the Gemini surfactants

**Fig 1:**
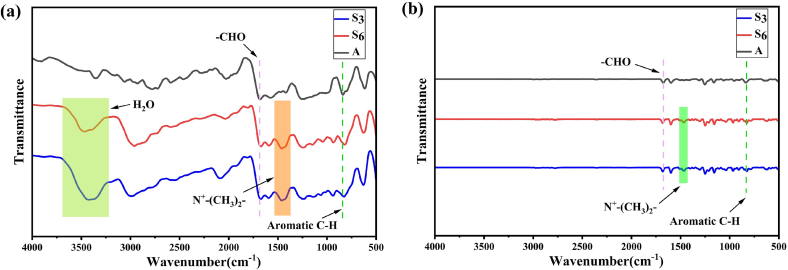
FT-IR spectra of A (4-(2-bromoethoxy) benzaldehyde), S_3_ and S_6_

The structure of the synthesized Gemini quaternary ammonium salt was confirmed by ^1^H NMR, ^13^C NMR, and MS analyses, as detailed in Section 2.2.2. Further verification was provided by FT-IR spectroscopy, with spectra of 4-(2-bromoethoxy) benzaldehyde, S_3_, and S_6_ shown in Fig. 1. Both the starting material and the final products exhibited characteristic bands at 1690 cm^-1^ and 830 cm^-1^, corresponding to the C=O stretching vibration of the aldehyde group and the aromatic C–H out-of-plane bending vibration of the para-substituted benzene ring, respectively. The persistence of these signals confirmed that the aldehyde functionality and the aromatic ring remained intact throughout the synthetic sequence.

In the spectra obtained via the KBr pellet method in Fig. 1(a), a broad absorption band between 3300 and 3600 cm^-1^ was observed, consistent with adsorbed water—a feature attributable to the hygroscopic nature of the ionic quaternary ammonium salts (S_3_ and S_6_). Notably, when analyzed using attenuated total reflectance (ATR) spectroscopy (Fig. 1(b)), this water-related band was absent, likely due to the surface-sensitive nature of ATR and reduced interference from ambient moisture. Despite the generally weaker signal intensity inherent to the ATR technique, the positions and assignments of the other key absorption bands remained consistent across both methods.

Crucially, the spectra of compounds S_3_ and S_6_ displayed a distinct pair of new bands at 1480 cm^-1^, characteristic of the asymmetric and symmetric bending vibrations of the N^+^–(CH₃) ₂– groups in the quaternary ammonium cation (Cheng, Xu, Wang, & Frost, 2016). This provides direct spectroscopic evidence for the successful formation of the target Gemini quaternary ammonium salt structure, fully consistent with the conclusions derived from the NMR and MS analyses in Section 2.2.2.

Imine bonds were readily formed when aldehyde-containing molecules encounter amine-containing molecules and six synthesized Gemini surfactants formed by the precursors S_3_ and S_6_ of two aldehyde containing quaternary ammonium salts and these primary amines were named S_3_- T_6_, S_3_- T_8_, S_3_- T_10_, S_6_- T_6_, S_6_- T_8_, S_6_- T_10_. The ^1^H NMR (Fig.S5, Fig.S6, Supplementary material) spectrum were adopted to confirm the formation of imine bonds. It showed that the peak area of the chemical shift around 9.8ppm which belongs to −CHO significantly decreases or even disappears, while the peak area at 8.2ppm which belongs to −CH=N− significantly appears. The conversion rate of imine bond can be obtained from the peak area ratio of them, and the conversion rate is shown in Table 1. The conversion rate of imine bond is at a high level (above 90%) and it can be considered that the majority of the mixture of primary amine and S_3_ or S_6_ in the system is the target Gemini surfactant product. In addition, other primary amines with long hydrophobic chains (dodecylamine, tetradecylamine, hexadecylamine) were used in the experiment. Because the chains were too long at room temperature, they would depolymerize soon after standing. Therefore, the amines with too long chains were not discussed in this paper.

**Table 1:** Conversion rate of imine bond in Gemini surfactant (different spacer lengths and different hydrophobic chain lengths)

Surfactants	S_3_ – T_6_	S_3_ – T_8_	S_3_ – T_10_	S_6_ – T_6_	S_6_ – T_8_	S_6_ – T_10_
Peak height at 9.8ppm	43.38	11.99	144.7	238.05	24.21	125.98
Peak height at 8.2ppm	657.44	1374.07	3055.46	1647.12	2351.6	3138.5
Conversion rate	93.4%	99.1%	95.3%	92.9%	99.0%	96.0%

### 3.2 Surface tension

**Fig 2:**
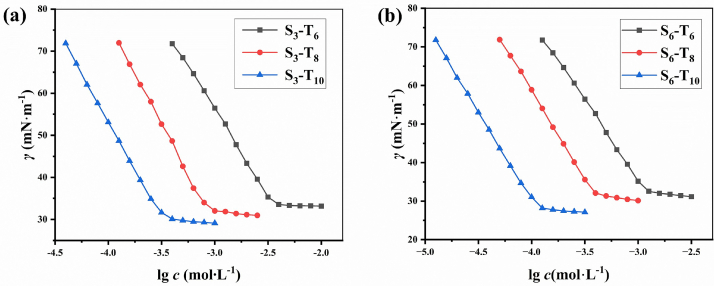
Plots of surface tension as a function of the logarithm of the concentration of Gemini surfactant based on S_3_ (a) and S_6_ (b) as a precursor at 25 °C

The surface activity of surfactants arises from the amphiphilic structure of their molecules. The hydrophilic groups of surfactants give the molecules a tendency to enter the water, and hydrophobic groups try to prevent them from solubilizing in the water and migrating outward from the interior of the water. Surfactants are generally adsorbed onto the air/liquid interface in an oriented fashion due to the unique molecular structures, resulting in decreasing the interfacial tension (Blokhuis, 2025; Shaban, Kang, & Kim, 2020; Zhao & Wang, 2017). As shown in Fig 2. (a) and Fig 2. (b), the equilibrium surface tension (*γ*) of the Gemini surfactants first underwent a rapid decrease with increasing the logarithm of the surfactant concentration and then tended toward a constant value, which is known as critical micelle concentration(*cmc)*. There is no minimum in either of the surface tension curves, ruling out the presence of other surface-active impurities. As the number of carbon atoms on the hydrophobic chain increases, the *cmc* significantly decreased and their *γ*_*cmc*_ also slightly decreased. The trend of decreasing *cmc* is similar to the Gemini surfactant products with reactant S_3_ and the products with reactant S_6_, but the *γ*_*cmc*_ of the S_6_ series products is lower. The possible reason for this is that the two head bases with the same charge in S_3_ are forced to be very close together, resulting in strong electrostatic repulsion. This repulsive force will make it difficult for molecules to closely arrange at the gas-liquid interface, leaving a large gap between molecules. The flexibility of S_6_ series Gemini surfactants allows it to bend or take a bent conformation. This reduces the electrostatic repulsion between the two head groups, and the hydrophobic chain can adjust its orientation more freely. Thus, the surfactant molecules can be arranged very closely at the interface to form highly ordered and dense monolayers. This can also be seen in the calculated maximum surface excess amount of the adsorbed surfactant (*Γ*_*max*_ Table 2): A larger *Γ*_*max*_ generally leads to a more compact air/liquid interfacial film possessing stronger effectiveness in reducing the surface tension (Adsorption of Surface-Active Agents at Interfaces, 2012).

**Table 2:** Surface activity parameters for synthesized Gemini surfactants at 25 °C

Parameters	S_3_ – T_6_	S_3_ – T_8_	S_3_ – T_10_	S_6_ – T_6_	S_6_ – T_8_	S_6_ – T_10_
*γ*_*cmc*_(mN·m^−1^)	34.32±0.076	32.73±0.083	30.99±0.043	33.41±0.049	32.29±0.055	28.88±0.062
*cmc* (mmol·L^−1^)	3.55±0.042^a^ 3.26±0.052^b^	0.912±0.026^a^ 0.963±0.067^b^	0.339±0.055^a^ 0.312±0.073^b^	1.20±0.053^a^ 1.13±0.081^b^	0.398±0.042^a^ 0.332±0.053^b^	0.117±0.019^a^ 0.103±0.026^b^
10^−6^*Γ*_*max*_ (mol·m^−2^)	3.45±0.062	3.58±0.078	3.84±0.077	3.64±0.056	3.84±0.063	3.87±0.082
*A*_min_(nm^2^)	0.481±0.045	0.464±0.038	0.432±0.013	0.456±0.062	0.432±0.056	0.429±0.044
*Π*_*cmc*_ (mN·m^−1^)	37.45±0.017	39.24±0.043	40.87±0.032	38.35±0.034	39.40±0.033	42.91±0.016
*pC*_*20*_	2.85±0.046	3.45±0.026	3.97±0.042	3.35±0.051	3.81±0.053	4.45±0.018

a Measured by surface tension method

b Measured by fluorescence experiment

Many properties of the surfactant solution undergo a dramatic change above the *cmc*. Thus, the *cmc* plays an important role in the practical applications. Similar to most straight-chain ionic surfactants, the *cmc* of the Gemini surfactants decreased to about one-third of the original upon increasing the hydrophobic tail by two –CH_2_– groups. Compared with 12-6-12, the *cmc* of S_6_- T_6_ is slightly larger and the *cmc* of S_6_- T_8_ is smaller (Brycki, Szulc, Brycka, & Kowalczyk, 2023). Therefore, it can be considered that the part containing benzene ring between the head group and imine bond in the Gemini surfactant is equivalent to 4-6 –CH_2_– groups. In contrast to the traditional surfactants, the Gemini surfactants synthesized in the experiment have better surface activity. For example, the *cmc* of S_6_- T_6_ is much smaller than that of traditional surfactant DTAB and it confirmed its strong micellization ability (Kuperkar, Modi, & Patel, 2012; Mivehi, Bordes, & Holmberg, 2011).

The surface performance of surfactants improves as the hydrophobic chain length increases when the connection groups were the same. Similarly, when the number of carbon atoms on the hydrophobic chain is same, *Γ*_*max*_ of the S_6_ series Gemini surfactants is larger than the S_3_ series, indicating the facial mask formed by S_6_ series surfactants are denser, it indicates that the facial mask it forms is denser. In addition, Gemini surfactants have larger *Γ*_*max*_ and denser arrangement at the air/water interface compared with traditional surfactants due to the unique structure.

The effectiveness in the γ reduction (*Π*_*cmc*_) can be obtained using Eq. (3):

(3) Πcmc=γ0−γcmc

where *γ*_*0*_ is the *γ* of pure water (71.93 mN·m^−1^). As listed in Table 2, the *Π*_*cmc*_ of S_3_- T_6_, S_3_- T_8_ and S_3_- T_10_ were relatively similar as a result of their similar *γ*_*cmc*_. Nonetheless, small differences still exist. Due to the same solvent-water, the *Π*_*cmc*_ shows an opposite change with that of the *γ*_*cmc*_. A larger *Π*_*cmc*_ indicates a stronger ability to decrease the *γ*. Therefore, S_3_- T_6_ is more effective in reducing the *γ* than S_3_- T_8_ and S_3_- T_10_. This is agreement with the conclusion deduced from the *γ*_*cmc*_ and *Γ*_*max*_. The pattern is also the same for S_6_ series Gemini surfactants and S_6_ series Gemini surfactant have a larger *Π*_*cmc*_ compared with S_3_ series when the lengths of hydrophobic chains are the same.

In addition, the adsorption efficiency of surfactants is typically evaluated using the negative logarithm of the surfactant concentration in the bulk phase required to reduce the solvent *γ* by 20 mN·m^−1^ (*pC*_*20*_) (Adsorption of Surface-Active Agents at Interfaces, 2012).

The larger the *pC*_*20*_, the higher the adsorption efficiency. As listed in Table 2, the *pC*_*20*_ of Gemini surfactants increases linearly with the increase in the number of carbon atoms in the hydrophobic tail, consistent with the results of conventional surfactants. Clearly, the longer the hydrophobic chain, the greater the driving force for migration to the interface, and the higher the adsorption efficiency.

### 3.3 Nile red fluorescence

Nile red is an oil soluble fluorescent probe with little fluorescence in water (Lazarus, Kothari, Venuganti, & Nag, 2025). However, while the micelles formed by Gemini surfactants, Nile red could accumulate in the hydrophobic interior, emitting fluorescence. Due to this characteristic, Nile red is usually used as a probe for the determination of *cmc*. The variation in the intensity of the Nile red fluorescence signal at 636 nm with the surfactant concentration was showed in Fig.S7. In general, the fluorescence intensity remained unchanged (∼0) below the critical concentration, indicating that micelles did not form and Nile red did not aggregate in the hydrophobic environment enclosed by micelles. Nonetheless, the fluorescence intensity began to increase rapidly above the critical concentration, which is usually interpreted as the dissolution of Nile red in the hydrophobic microenvironment. That is to say, micelles are formed. Therefore, the critical concentration corresponds to *cmc*. As shown in Table 2, *cmc* measured from the change of fluorescence intensity of Nile red is close to *cmc* measured by surface tension method, further verifying the accuracy of the surface tension measurement.

### 3.4 Conductivity

**Fig 3:**
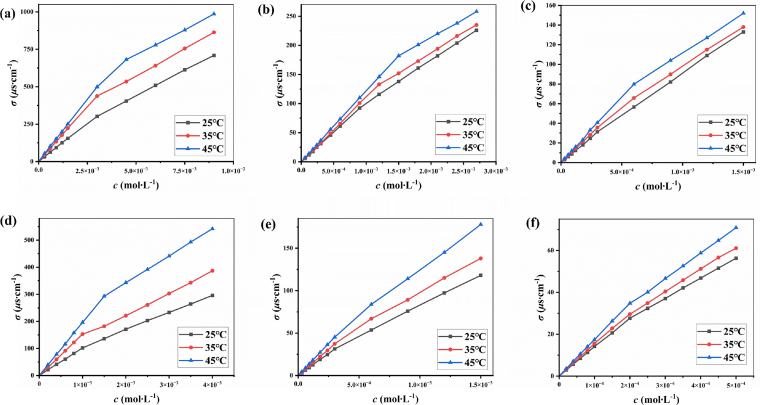
Plotting the conductivity as a function of the concentration of S_3_- T_6_ (a), S_3_- T_8_ (b), S_3_- T_10_ (c), S_6_- T_6_ (d), S_6_- T_8_ (e) and S_6_- T_10_ (f) at different temperatures.

At 25°C, 35°C and 45 °C, the conductivity (*σ*) of the Gemini surfactant solutions was measured respectively. From the results exhibited in Fig 3, *σ* increased linearly at both low and high concentrations, but the change in *σ* is not monotonous. Due to the transformation of conductive substances, the change in *σ* is more pronounced at low concentrations than at high concentrations. Namely, the *σ-c* curve can be divided into two linear segments by a breakpoint, corresponding to the critical micelle concentration (*cmc*). The *cmc* of Gemini surfactants measured by conductivity method is shown in Table 3, which is similar to that measured by surface tension method and fluorescence experiment. Normally, heating accelerates the migration of conductive ions in the solution, resulting in higher conductivity. Therefore, the slopes of linear segments also increases as the temperature increasing, and the breakpoint shifts to a higher concentration, reflecting a larger *cmc (*Bamyani & Bagheri, 2025*;* Savle et al., *2025**)*.

**Table 3:** The *cmc* via conductivity measurements of Gemini surfactants at different temperatures

Surfactant	S_3_ – T_6_	S_3_ – T_8_	S_3_ – T_10_	S_6_ – T_6_	S_6_ – T_8_	S_6_ – T_10_
*cmc* at 25 °C(mmol·L^−1^)	3.03± 0.059	0.977± 0.062	0.307± 0.038	1.16± 0.054	0.324± 0.065	0.118± 0.015
*cmc* at 35 °C(mmol·L^−1^)	3.36± 0.044	1.21± 0.051	0.423± 0.033	1.28± 0.065	0.395± 0.056	0.143± 0.062
*cmc* at 45 °C(mmol·L^−1^)	3.87± 0.029	1.47± 0.049	0.558± 0.046	1.47± 0.063	0.477± 0.074	0.198± 0.054
1-*β* at 25 °C	0.608± 0.060	0.715± 0.055	0.798± 0.061	0.633± 0.028	0.753± 0.033	0.818± 0.054
1-*β* at 35 °C	0.526± 0.055	0.616± 0.077	0.713± 0.048	0.576± 0.043	0.696± 0.061	0.743± 0.042
1-*β* at 45 °C	0.423± 0.052	0.525± 0.044	0.603± 0.041	0.515± 0.026	0.613± 0.039	0.703± 0.051

The *σ-c* curve consists of two linear segments. In Fig 3, the ratio of the slopes of the upper and lower linear segments of *cmc* is usually defined as the ionization degree (*β*).Therefore, 1-*β* is defined as the binding degree of counterions(Br^-^) of ionic surfactants to micelles formed by combining hydrophilic positive quaternary ammonium cations (Hantal, Sega, Horvai, & Jedlovszky, 2021; Lunkenheimer, Prescher, & Geggel, 2022; Tiwari, Namsani, & Singh, 2022). As shown in Table 3, the 1-*β* value increases with the increase of hydrophobic chain length, which is mainly due to the enhanced hydrophobicity and micellization of Gemini surfactants with the increase of hydrophobic chain length. On the other hand, the micelles formed by surfactants are small and loose when the hydrophobic chain is short, the electrostatic environment on the surface of micelles was relatively "open", leading the counterion binding not firm and low degree of binding. Accordingly, the growth of hydrophobic chain formed tighter micelles and enhance electrostatic interaction, so as to combine counterions with micelles.

When the hydrophobic chain length is the same, the 1- *β* value of series S_3_ Gemini surfactants is lower than that of series S_6_. The shorter the linker between two hydrophilic groups, the closer the two head groups with the same charge are forced to come together，resulting in greater electrostatic repulsion. The head groups are arranged relatively loosely on the surface of the micelles and are far away from each other in order to reduce the repulsion, leading the charge density on the surface of the micelles to be low. Therefore, the "grasping" ability of counterions is weak and the binding degree of counterions is low. On the other hand, the longer linking groups increased flexibility of S_6_ Gemini surfactants. The head groups can adjust their positions more freely by bending the electrostatic pressure between them, to form closer arrangement and higher degree of counterion binding on the surface of the micelles. This reflects the change of micelle structure, accompanied by more efficient counterion binding indirectly. It can also be seen from their A_min_ that the larger A_min_ confirms the smaller 1- *β* indirectly

Moreover, it can be seen from Table 3 that temperature also has some impact on 1- *β*. Thermal motion of counterions increases with the increase of temperature, and more kinetic energy breaks away from the binding is the reason for this phenomenon (Edwards & Williams, 2004; Sowada, 1994). Other ionic surfactants also have similar phenomenon. The morphology of micelles may change as the degree of binding between micelles and counterions becomes lower.

### 3.5 Micromorphology and self-assembling structure of Gemini surfactants

As the concentration of Gemini surfactant aqueous solutions increased, a marked decrease in transmittance was observed, accompanied by the formation of an opaque, milky emulsion. This visual change suggested the onset of aggregation beyond simple micelles (Stancheva, Georgiev, Radulova, Danov, & Marinova, 2022). At low concentrations, Gemini surfactants typically assemble into micelles with diameters around 10 nm. With increasing concentration, these molecules reorganize into larger structures such as vesicles (∼100 nm in diameter) featuring bilayer membranes, or worm-like micelles (Dhanagar & Shaheen, 2025; Kelleppan et al., 2018; Li, Zheng, Zhao, Pang, & Wang, 2025).

**Fig 4:**
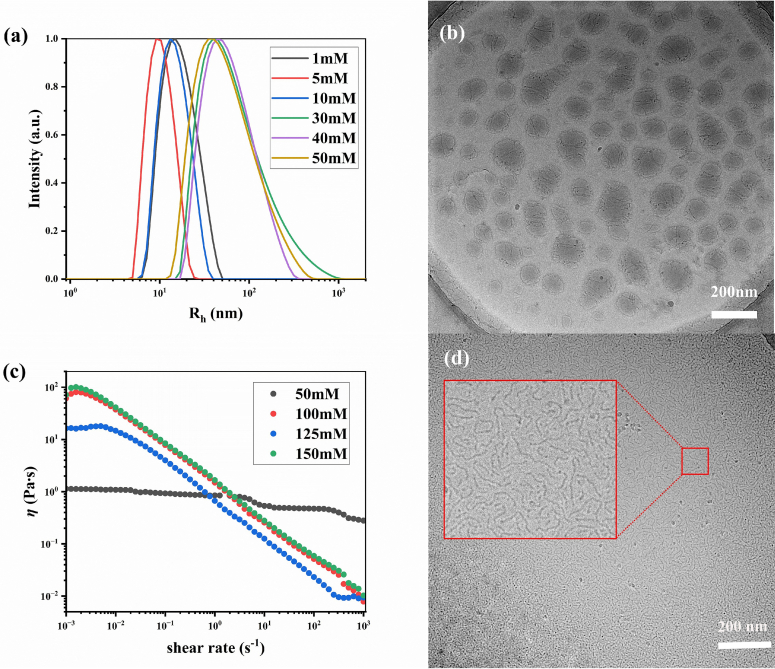
Particle size distribution(a), Cryo-TEM of vesicles(b), steady-state rheological curve(c), Cryo-TEM of worm-like micelles (d) of S_6_-T_10_ aqueous solution at different concentrations

Macroscopically, a Tyndall effect was clearly observed in the aqueous solution of S_6_-T_10_ (Fig.S8, Supplementary Material) upon beam illumination, indicating the presence of vesicles in the 50 mmol·L^-1^ surfactant solution. From a microscopic perspective, dynamic light scattering (DLS) revealed that the hydrodynamic diameter of the aggregates was approximately 10 nm when the surfactant concentration ranged between 1 and 10 mmol·L^-1^ (Fig. 4(a)). As the concentration increased to 30–50 mmol·L^-1^, the particle size increased sharply to about 100 nm—an order of magnitude larger—suggesting a morphological transition to non-micellar aggregates. Cryo-TEM imaging (Fig. 4(b)) confirmed the presence of vesicles in solution. Additionally, a number of granular entities were observed in the micrographs, which are presumed to be precursor particles of the Gemini quaternary ammonium salts that had not been fully converted into the target surfactants (Section 3.1).

With further increases in surfactant concentration, the solution transparency decreased drastically, rendering DLS analysis infeasible. Therefore, rheological behavior was investigated (Fig. 4(c)). At 50 mmol·L^-1^, the viscosity remained relatively stable under varying shear rates. In contrast, at higher concentrations, a pronounced shear-thinning behavior was observed, with the viscosity dropping by two to three orders of magnitude from low to high shear rates—a characteristic signature of worm-like micelle formation (Kusano, Oyama, Yoshida, & Tanaka, 2025; Parathakkatt, Gokul, & Markandewar, 2025; Zhang et al., 2025). The presence of such worm-like micelles was directly visualized via Cryo-TEM (Fig. 4(d)).

### 3.6 Studies on pH responsiveness of surfactants and controllable release of curcumin

#### 3.6.1 Macroscopic phenomenon

As a class of dynamic covalent bonds, imine bonds—formed from aldehyde-containing quaternary ammonium salts and primary amines—exhibit stability under alkaline conditions and cleave under acidic conditions, enabling reversible formation and decomposition of Gemini surfactants via pH adjustments. In the Nile Red experiment in 3.3, the mixed solution appeared pink while surfactants are existed. Differently, Nile red did not show any color in pure water. The surfactant solution, after being mixed with Nile Red, exhibited distinct colors at different pH values (Fig. S9, Supplementary Material). Therefore, the presence or absence of surfactants can be inferred from the solution color, providing indirect evidence that the synthesized surfactants are pH-sensitive.

On the other hand, when its concentration was much higher than the *cmc*, the S_6_–T_10_ solution became opaque, because the Gemini surfactants self-assembled into worm-like micelles in an alkaline environment (Fig.S10(a), Supplementary Material). Correspondingly, the imine bonds were cleaved under acidic conditions, so no Gemini surfactants remained and the solution stayed transparent. Moreover, abundant foam was generated upon shaking the S_6_–T_8_ solution near its *cmc* at pH 8, and the foam remained stable after 30 min of standing (Fig.S10(b), Fig.S10(c), Supplementary Material). A small amount of foam was observed in the same sample after shaking at pH 5. This foaming resulted from the reaction between a trace amount of hydrophilic aldehyde-containing precursor and the primary amine, which generated surfactants. However, this was a transitional state, as the Gemini quaternary ammonium salts and the primary amines separated after the sample was left to stand. This phenomenon macroscopically demonstrates the pH-sensitive nature of the Gemini surfactants.

#### 3.6.2 UV–visible spectroscopic studies

**Fig 5:**
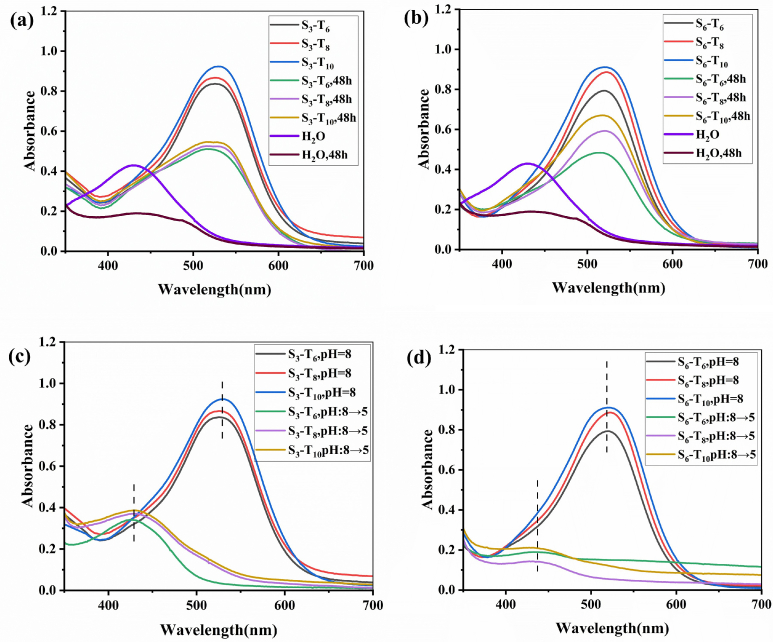
UV-visible spectra of S_3_-Curcumin(a), S_6_-Curcumin(b), S_3_-Curcumin at different pHs(c) and S_6_-Curcumin at different pHs(d).

The solubility of curcumin in the presence of Gemini surfactants was determined using UV–visible spectroscopy. The concentrations of the S_3_ and S_6_ surfactant solutions were fixed at 5 times their respective *cmc* values. As shown in Fig. 5(a) and Fig. 5(b), the S_3_-curcumin and S_6_-curcumin systems exhibited maximum absorbance at 526 nm and 519 nm, respectively. The apparent solubility of curcumin increased slightly with hydrophobic chain length in both the S_3_ and S_6_ Gemini surfactant series (with the effect being more significant in S_6_). This can be explained by the rigid structure of these surfactants. The short connecting group forces the two hydrophobic chains into close, parallel, and tight packing, which significantly limits the conformational freedom of the molecules. Furthermore, the steric hindrance from the benzene ring attached to the hydrophilic group additionally restricts conformational transitions, making it more difficult for the surfactants to solubilize curcumin effectively.

The retention of curcumin encapsulated by the Gemini surfactants was measured after 48 hours. Data in Table 4 indicate that the encapsulation efficiency was approximately 60% for the S_3_ series and 60-75% for the S_6_ series. The superior performance of S_6_-T_10_, which possesses the longest linker and tail, is ascribed to the formation of more robust micelles featuring an expanded hydrophobic core and enhanced stability.

**Table 4:** The residual rate of curcumin (48 h) in different Gemini surfactants aqueous solutions

Surfactant	S_3_ – T_6_	S_3_ – T_8_	S_3_ – T_10_	S_6_ – T_6_	S_6_ – T_8_	S_6_ – T_10_
Residual rate (%)	60.9	60.6	59.7	61.3	70.1	73.6

The dynamic pH-responsiveness, governed by the imine bond, was verified by reducing the pH from 8 to 5 as shown in Fig. 5(c) and Fig. 5(d). This stimulus induced a rapid decline in the characteristic UV-vis absorption of curcumin, signifying its prompt release. This phenomenon results from the acid hydrolysis of the imine bonds, which dismantles the micellar architecture and releases the payload. A concomitant blue shift in the absorption maximum was also observed, arising from the protonation of curcumin's phenolic groups under acidic conditions, which alters its electronic conjugation.

#### 3.6.3 Fluorescence spectroscopic studies

**Fig 6:**
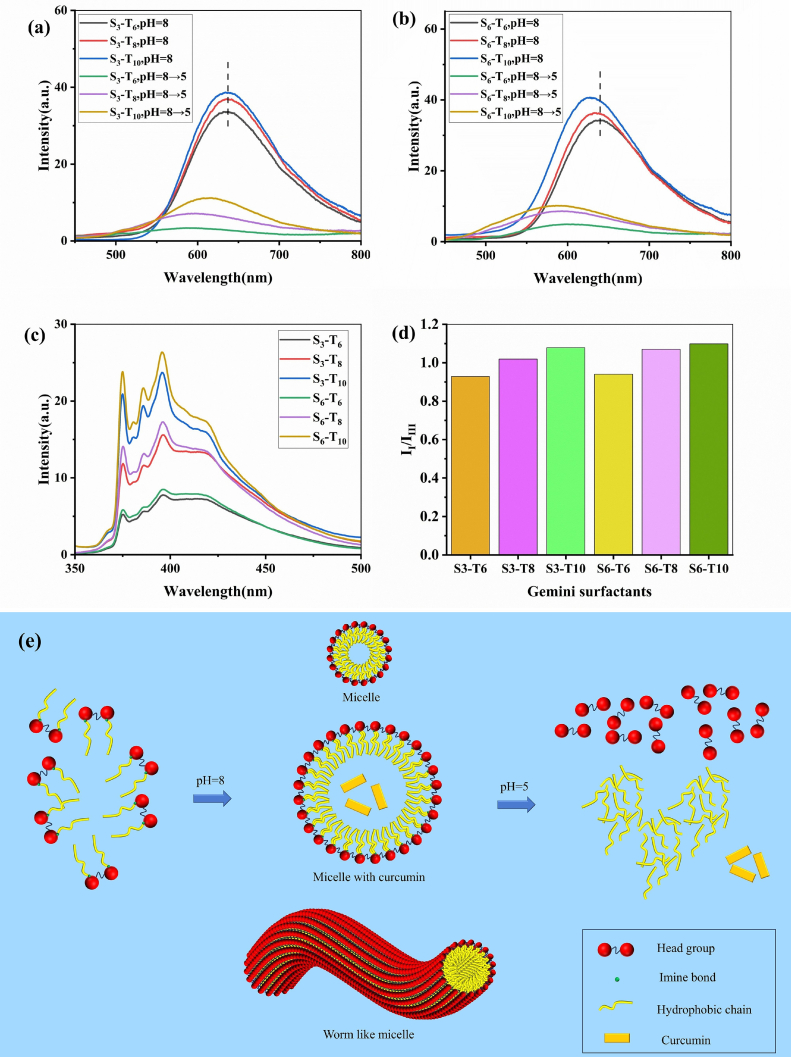
Fluorescence spectra of S_3_-Curcumin at different pHs(a), S_6_-Curcumin at different pHs(b), pyrene(c) and I_I_/I_III_ of pyrene(d), schematic diagram of self-assembly of Gemini surfactants and controlled release of curcumin(e).

The fluorescence spectroscopy of curcumin revealed a broad emission peak at 553 nm in aqueous solution. Upon interaction with S_3_ and S_6_ series Gemini surfactants, the fluorescence intensity of curcumin was significantly enhanced. As shown in Fig. 6(a) and Fig. 6(b), the maximum emission wavelengths for the S_3_-curcumin and S_6_-curcumin systems were located at 637 nm and 629 nm, respectively. With increasing hydrophobic chain length, the emission peak of S_3_–curcumin remained largely unchanged. In contrast, a pronounced blue shift was observed for S_6_-curcumin under the same condition.

This divergence can be attributed to the differential influence of the linker rigidity relative to the hydrophobic chain length. For S_3_ surfactants, the short and rigid linker dominates the microenvironment around curcumin, thereby masking the effect of hydrophobic chain elongation and resulting in minimal change in local polarity. In the case of S_6_ surfactants, however, the longer and more flexible linker allows the growing hydrophobic chain to effectively reduce the polarity of the micellar core. As a result, curcumin partitions into a more hydrophobic region, leading to the marked blue shift in its fluorescence emission. This interpretation aligns with the UV–vis spectroscopic results discussed in section 3.6.2.

Furthermore, when the environmental pH was lowered, a sharp decrease in the fluorescence emission intensity of curcumin was observed, corroborating the excellent pH-responsive behavior of the Gemini surfactants.

Pyrene, a polycyclic aromatic hydrocarbon widely employed as a fluorescent probe, exhibits several well-defined fluorescence emission bands at approximately 375 nm (Band I), 379 nm (Band II), 385 nm (Band III), 395 nm (Band IV), and 410 nm (Band V). The intensity ratio of Band I to Band III (I_I_/I_III_) is highly sensitive to the polarity of its immediate microenvironment (Ayyavoo & Velusamy, 2021). In hydrophilic environments, the intensity of Band I is stronger than that of Band III, whereas this trend reverses under nonpolar conditions. When surfactant molecules aggregate into micelles, pyrene is typically solubilized within the hydrophobic core or anchored at the palisade layer.

In our experiments, the I_I_/I_III_ ratio of pyrene in pure water was measured as 1.81. As illustrated in Fig. 6(c) and Fig. 6(d), the I_I_/I_III_ value in the surfactant-curcumin system decreased to around 1.0, indicating that curcumin is encapsulated in a nonpolar region. Furthermore, it was observed that shorter hydrophobic chains led to even lower I₁/I₃ values. This trend may be attributed to the formation of more uniform and compact hydrophobic microdomains by shorter-chain surfactants, which more effectively exclude water molecules and thus provide a less polar environment for the pyrene probe. Furthermore, the self-assembly of Gemini surfactants and controlled release of curcumin are shown in Fig. 6(e). The synthesized Gemini surfactants self-assemble into micellar aggregates (e.g., spherical or worm-like micelles) in alkaline aqueous solution, which effectively encapsulate curcumin. Under acidic conditions, however, the cleavage of imine bonds causes the dissociation of the hydrophilic head and hydrophobic tail, disrupting the micellar structure and thereby releasing the encapsulated curcumin. This demonstrates the excellent pH-responsiveness of the surfactants.

## 4 Conclusions

In summary, this work presents the successful synthesis and comprehensive characterization of a new family of pH-responsive Gemini surfactants based on hydrolyzable imine bonds. The synthesized compounds, obtained with high precursor conversion (>90%), demonstrated outstanding surface activity. A structure-property relationship analysis confirmed that their surface activity and counterion binding affinity are critically governed by the molecular geometry, i.e., the length of the hydrophobic alkyl chain and the spacer group. The S_6_ surfactant series was identified as optimal, with longer chains generally conferring enhanced performance. Concurrently, the self-assembly morphology was observed to evolve from micelles to vesicles and worm-like micelles with increasing concentration. The pivotal pH-sensitive cleavage of the imine bond was unequivocally demonstrated by UV-Vis and fluorescence spectroscopy, a property leveraged for the controlled encapsulation and release of the model drug curcumin. Collectively, these results not only elucidate key structure–function relationships in stimuli-responsive surfactant systems, but also highlight their promising utility in targeted drug delivery, smart material design, and other biomedical applications where on-demand release is essential. This work thus provides both fundamental insights and practical pathways toward the development of next-generation responsive soft materials.

## CRediT authorship contribution statement

**Wenbo Zhao:** Writing – original draft, Methodology, Conceptualization. **Heng Zhang:** Writing – review & editing, Formal analysis, Conceptualization. **Wenwen Yu:** Writing – review & editing, Investigation. **Fengbo Zhu:** Data curation, Conceptualization. **Jianjun Xu:** Resources, Funding acquisition. **Quanxin Xu:** Software, Funding acquisition. **Hongwei He:** Software. **Fuyong Liu:** Software. **Qiang Zheng:** Resources, Methodology.

## Declaration of competing interest

The authors declare that they have no known competing financial interests or personal relationships that could have appeared to influence the work reported in this paper.

## Data Availability

Data will be made available on request.

## Appendix A Supplementary data

Supplementary material: It includes the following figures: Fig. S1: ¹H NMR and ¹³C NMR spectra of the intermediate 4-(2-bromoethoxy)benzaldehyde. Fig. S2:¹H NMR and ¹³C NMR spectra of the synthesized Gemini surfactant precursors S₃ and S₆. Fig. S3: High-resolution mass spectrometry (HRMS) data confirming the molecular weight of S₃ and S₆. Fig. S4: Surface tension measurements of water and the Gemini surfactant solutions for critical micelle concentration (cmc) determination. Fig. S5 & S6: ¹H NMR spectra demonstrating the dynamic imine bond formation between surfactants (S₃, S₆) and various tail amines (T₆, T₈, T₁₀). Fig. S7: Fluorescence intensity plots used to determine the cmc of S₃- and S₆-based surfactants. Fig. S8: Visual evidence (transparency and Tyndall effect) of micelle formation in S₆-T₈ solutions. Fig. S9: Visual demonstration of the pH-responsive behavior of the surfactant assemblies. Fig. S10: Photos illustrating the solubility and solution stability of selected surfactants. Image 1

## References

[bb0005] Adsorption of Surface-Active Agents at Interfaces (2012).

[bb0010] Ayyavoo K., Velusamy P. (2021). Pyrene based materials as fluorescent probes in chemical and biological fields. New Journal of Chemistry.

[bb0015] Bamyani N., Bagheri A. (2025). Interactions between cationic surfactant and poly ethylene glycol: Effect of the polymer concentration and alkyl chain length of surfactant. Journal of Molecular Liquids.

[bb0020] Blokhuis E.M. (2025). Quantitative description of the surface tension minimum in a two-component surfactant system. Langmuir.

[bb0025] Brycki B., Szulc A., Brycka J., Kowalczyk I. (2023). Properties and applications of quaternary ammonium gemini surfactant 12-6-12: An overview. Molecules.

[bb0030] Cheng H., Xu P., Wang D., Frost R.L. (2016). Thermal decomposition behavior and de-intercalation kinetics of kaolinite/quaternary ammonium salt complexes. Journal of Thermal Analysis and Calorimetry.

[bb0035] Dhanagar A., Shaheen A. (2025). Self-assembled vesicles from carbon quantum dots induced by a gemini surfactant for selective detection of picric acid. Langmuir.

[bb0040] Edwards S.A., Williams D.R.M. (2004). Hofmeister effects in colloid science and biology explained by dispersion forces: analytic results for the double layer interaction. Current Opinion in Colloid & Interface Science.

[bb0045] Fan Q., Tang Y., Sun H., Guo D., Ma J., Guo J. (2024). Cluster-triggered self-luminescence, rapid self-healing, and adaptive reprogramming liquid crystal elastomers enabled by dynamic imine bond. Advanced Materials.

[bb0050] Fidalgo A.C.D., Costa M.A.M., Garcia-Rìo L., Gerola A.P. (2025). A functional polymer/surfactant nanoassembly for pH-responsive delivery of curcumin. Journal of Molecular Liquids.

[bb0055] Ghosh A., Seth S.K., Purkayastha P. (2018). Surfactant and cyclodextrin induced vesicle to micelle to vesicle transformation in aqueous medium. Langmuir.

[bb0060] Guo R., Qi W., Liu H., Li D., Chen G., Li Q., Zhou Z. (2024). Dynamic borate ester bond-based 3D printing fluorescence polysiloxane with self-healing, antimicrobial, and shape memory. Chemical Engineering Journal.

[bb0065] Hantal G., Sega M., Horvai G., Jedlovszky P. (2021). Contribution of different molecules and moieties to the surface tension in aqueous surfactant solutions. II: Role of the size and charge sign of the counterions. The Journal of Physical Chemistry B.

[bb0070] Hu B., Liu J., Deng C., Xing Y., Gong M., Wu Z., Wang G. (2025). Multifunctional pH-Responsive gemini surfactant. Small.

[bb0075] Kelleppan V.T., Moore J.E., McCoy T.M., Sokolova A.V., Campo L.D., Wilkinson B.L., Tabor R.F. (2018). Self-assembly of long-chain betaine surfactants: Effect of tailgroup structure on wormlike micelle formation. Langmuir.

[bb0080] Kim J., Choi J., Park H., Lee J., Lee H.-J., Park J.D., Lee J.B., Na Y., Yoon C. (2025). Stimuli-responsive biodegradable soft gripper composed of pH-responsive alginate/gelatin/acrylic acid and non-pH-responsive acrylamide bilayer. European Polymer Journal.

[bb0085] Kuang J., Gao J., Xie S., Lei Q., Fang W., Xie H., Lu X. (2020). Phase behaviors and curcumin encapsulation performance of Gemini surfactant microemulsion. Journal of Molecular Liquids.

[bb0090] Kuperkar K., Modi J., Patel K. (2012). Surface-active properties and antimicrobial study of conventional cationic and synthesized symmetrical gemini surfactants. Journal of Surfactants and Detergents.

[bb0095] Kusano T., Oyama N., Yoshida H., Tanaka H. (2025). Varying the rheological behavior of a micellar solution via modified microscopic structures in the presence of graphene oxide. RSC Advances.

[bb0100] Lazarus R., Kothari R., Venuganti V.V.K., Nag A. (2025). Intracellular temperature sensing with remarkably high relative sensitivity using nile red-loaded biocompatible niosome. ACS Applied Bio Materials.

[bb0105] Le Bastart P., de Villeneuve F., Trummer N., Preisig H., Yalcinkaya J., Venzmer J., Sottmann C.S. (2025). Self-assembly and liquid crystalline phases of the biosurfactant di-rhamnolipid. Journal of Molecular Liquids.

[bb0110] Li G., Zheng Z., Zhao X., Pang X., Wang K. (2025). The effect of asymmetry on the surface properties and aggregation behavior of asymmetric Gemini Surfactants. Journal of Molecular Structure.

[bb0115] Li H., Liu X. (2022). Rational design of dynamic imine surfactants for oil–water emulsions: Learning from oil-induced reversible dynamic imine bond formation. Journal of Colloid and Interface Science.

[bb0120] Li Q., Zhang Y., Zheng X., Qi J., Wu Y., Zhang Z., Lu H. (2025). Construction of a pH-switchable surfactant with multiple response sites for rapid release of emulsion drag reducer. Langmuir.

[bb0125] Liu X., Yu M. (2025). Synergistic effect of gemini cationic/anionic surfactant mixtures for enhanced oil recovery. Energy & Fuels.

[bb0130] Liu Y., Wang T., Wang W. (2025). Photopharmacology and photoresponsive drug delivery. Chemical Society Reviews.

[bb0135] Lu P., He S., Zhou Y., Zhang Y. (2021). Adsorption, micellization and antimicrobial activity of formyl-containing cationic surfactant in diluted aqueous solutions. Journal of Molecular Liquids.

[bb0140] Lu S., Liu Y., Dong J., Li X. (2025). Dilution-driven gel-sol-gel-sol transitions by the sequential evolution of surfactant micelles. Nature Communications.

[bb0145] Lunkenheimer K., Prescher D., Geggel K. (2022). Role of counterions in the adsorption and micellization behavior of 1:1 ionic surfactants at fluid interfaces—demonstrated by the standard amphiphile system of alkali perfluoro-n-octanoates. Langmuir.

[bb0150] Mivehi L., Bordes R., Holmberg K. (2011). Adsorption of cationic gemini surfactants at solid surfaces studied by QCM-D and SPR: Effect of the rigidity of the spacer. Langmuir.

[bb0155] Nisoa M., Kaewpradit S., Nahar L., Sarker S.D., Charoensup R., Puttarak P., Yusakul G. (2025). Extraction of curcumin and curcuminoids: From conventional methods to innovative extraction using deep eutectic solvents. Microchemical Journal.

[bb0160] Parathakkatt S., Gokul G.K., Markandewar R.A. (2025). Microstructural evolution in DTAB/KBr micellar systems induced by nonanol: A rheological perspective. Journal of Molecular Liquids.

[bb0165] Peng F., Chen Y., Liu J., Xing Z., Fan J., Zhang W., Qiu F. (2021). Facile design of gemini surfactant-like peptide for hydrophobic drug delivery and antimicrobial activity. Journal of Colloid and Interface Science.

[bb0170] Pinthong T., Yooyod M., Mahasaranon S., Viyoch J., Jongjitwimol J., Ross S., Ross G. (2025). Designing stable macroporous hydrogels: Effects of single and dual surfactant systems on porous architecture, absorption capacity, and mechanical strength. ACS Applied Polymer Materials.

[bb0175] Prajapati V., Mata J., Hoare J.G., Christie L.D., Singer R.D., Marangoni D.G., Kuperkar K., Bahadur P. (2025). Micelle morphology modulation in structurally diverse polyoxyethylene-based nonionic surfactants in the presence of cationic gemini surfactants: An in-depth scattering analysis. Journal of Molecular Liquids.

[bb0180] Qiao M., Fan J., Ding L., Fang Y. (2021). Fluorescent ensemble sensors and arrays based on surfactant aggregates encapsulating pyrene-derived fluorophores for differentiation applications. ACS Applied Materials & Interfaces.

[bb0185] Rai M., Feitosa C.M., Ingle A.P., Golinska P. (2025). Harnessing bioactive nanocurcumin and curcumin nanocomposites to combat microbial pathogens: a comprehensive review. Critical Reviews in Biotechnology.

[bb0190] Rajput S.M., Mondal K., Kuddushi M., Jain M., Ray D., Aswal V.K., Malek N.I. (2020). Formation of hydrotropic drug/gemini surfactant based catanionic vesicles as efficient nano drug delivery vehicles. Colloid and Interface Science Communications.

[bb0195] Sanchez-Huerta C., Zhang S., Alahmari M., Humam A.A., Hong P.-Y. (2025). Remediation of petroleum hydrocarbons in contaminated groundwater with the use of surfactants and biosurfactants. Chemosphere.

[bb0200] Savle A.M., Mishra-Kadam N., Girase M., Dubey D.M., Chourasiya K.R., Kuperkar K.C., Kadam Y.K. (2025). Impact of the BCS class-II antihypertensive drug Irbesartan on the micellization behaviour of cationic surfactant DTAB: Insights from conductometry, surface tension, UV–visible spectroscopy and computational studies. Journal of Molecular Liquids.

[bb0205] Shaban S.M., Kang J., Kim D.-H. (2020). Surfactants: Recent advances and their applications. Composites Communications.

[bb0210] Sokjorhor J., Phantan C., Ratanathawornkit K., Crespy D. (2025). Simultaneous self-healing and corrosion protection using disulfide bonds. Advanced Functional Materials.

[bb0215] Sowada R. (1994). The effect of electrolytes on the critical micelle concentration of tonic surfactants. The Corrin-Harkins equation.

[bb0220] Stancheva T.N., Georgiev M.T., Radulova G.M., Danov K.D., Marinova K.G. (2022). Rheology of saturated micellar networks: Wormlike micellar solutions vs. bicontinuous micellar phases. Colloids and Surfaces A: Physicochemical and Engineering Aspects.

[bb0225] Tiwari S., Namsani S., Singh J.K. (2022). Effect of salt on the adsorption of ionic surfactants at the air-water interface. Journal of Molecular Liquids.

[bb0230] Turk M., Kogej K., Hansson P. (2025). Interaction of sodium polystyrenesulfonate with fluorinated ionic surfactant of opposite charge. Langmuir.

[bb0235] Varytimiadou S., Giesselmann F. (2025). Viscoelastic properties of micellar lyotropic nematic liquid crystals: exploring the impact of temperature and surfactant concentration. Langmuir.

[bb0240] Wang W., Ge J., Chu P., Zhang Z., Xu S., Lv J. (2025). Wormlike micelles induced by diverse metal ions in anionic/zwitterionic binary surfactant systems. Journal of Molecular Liquids.

[bb0245] Liu Y.-S., Yan B. (2025). Exciton regulation and fluorescence switching via redox-responsive heterojunction interfaces. Angewandte Chemie International Edition.

[bb0250] Yang S., Park S., Kim S., Kim S.-K. (2024). Vitrimer with dynamic imine bonds as a solid-state electrolyte for lithium metal batteries. Materials Today Energy.

[bb0255] Yi T., Lu Y., Chen B., Wang X., Zhang M., Frostad J.M., Xu Z. (2026). Pseudo-Gemini surfactants as switchable surfactants for foams. Colloids and Surfaces A: Physicochemical and Engineering Aspects.

[bb0260] Zhang K.-X., Ding A.-X., Tan Z.-L., Shi Y.-D., Lu Z.-L., He L. (2018). Tetraphenylethylene-based gemini surfactant as nonviral gene delivery system: DNA complexation, gene transfection and cellular tracking. Journal of Photochemistry and Photobiology A: Chemistry.

[bb0265] Zhang W., Gao J., Duan X., Li D., Chen H., Chen L., Zhang Q. (2025). Rheological behavior and mechanism of pH-responsive wormlike micelles based on Sodium cocoyl glycinate/cocamidopropyl hydroxypropyl sulfobetaine. Colloids and Surfaces A: Physicochemical and Engineering Aspects.

[bb0270] Zhao J., Jia W., Zhang R., Wang X., Zhang L. (2024). Improving curcumin bioavailability: Targeted delivery of curcumin and loading systems in intestinal inflammation. Food Research International.

[bb0275] Zhao W., Wang Y. (2017). Coacervation with surfactants: From single-chain surfactants to gemini surfactants. Advances in Colloid and Interface Science.

