# Supplementary material for: From self-assembly to controlled release: synthesis and properties of dynamic imine-based Gemini surfactants for curcumin delivery
Source: Food Chem X. 2026 Feb 8;34:103635. doi: 10.1016/j.fochx.2026.103635 (PMC12914832; doi:10.1016/j.fochx.2026.103635)
Supplement: Supplementary file 1 — Supplementary material: It includes the following figures: Fig. S1: ¹H NMR and ¹³C NMR spectra of the intermediate 4-(2-bromoethoxy)benzaldehyde. Fig. S2:¹H NMR and ¹³C NMR spectra of the synthesized Gemini surfactant precursors S₃ and S₆. Fig. S3: High-resolution mass spectrometry (HRMS) data confirming the molecular weight of S₃ and S₆. Fig. S4: Surface tension measurements of water and the Gemini surfactant solutions for critical micelle concentration (cmc) determination. Fig. S5 & S6: ¹H NMR spectra demonstrating the dynamic imine bond formation between surfactants (S₃, S₆) and various tail amines (T₆, T₈, T₁₀). Fig. S7: Fluorescence intensity plots used to determine the cmc of S₃- and S₆-based surfactants. Fig. S8: Visual evidence (transparency and Tyndall effect) of micelle formation in S₆-T₈ solutions. Fig. S9: Visual demonstration of the pH-responsive behavior of the surfactant assemblies. Fig. S10: Photos illustrating the solubility and solution stability of selected surfactants. [file mmc1.docx]

***Supplementary material***

**From self-assembly to controlled release: synthesis and properties of dynamic imine-based Gemini surfactants for curcumin delivery**

**Wenbo Zhao^a^, Heng Zhang^a, *^, Wenwen Yu^a, *^, Fengbo Zhu^a^, Jianjun Xu^b^, Quanxin Xu^b^, Hongwei He^a^, Fuyong Liu^a^ and Qiang Zheng^a,c, *^**

*^a^ College of Materials Science and Engineering, Taiyuan University of Technology, Taiyuan 030024, China ;*

*^b^ R&D Center, XiYueFa International Environmental Protection New Material Co., Ltd, Taiyuan 030006, China;*

*^c^ Department of Polymer Science & Engineering, Zhejiang University, Hangzhou 310027, China*

*E-mail: zhangheng@tyut.edu.cn (H.Z.); yuwenwen@tyut.edu.cn (W.Y.); zhengqiang@zju.edu.cn (Q.Z.)*


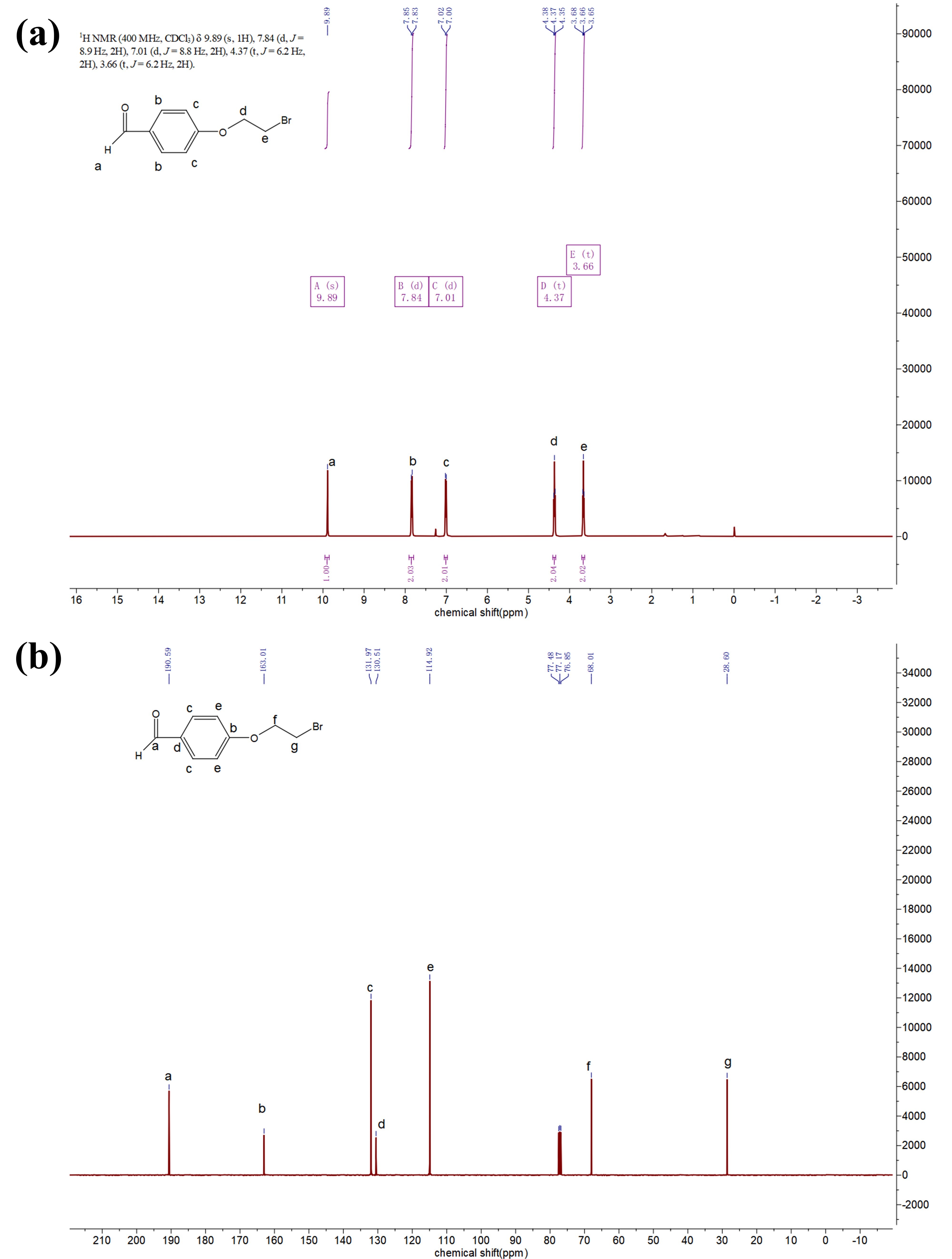


**Fig. S1.** ^1^H NMR spectrum (a) and ^13^C NMR spectrum (b) of 4-(2-bromoethoxy) benzaldehyde using CDCl_3_ as solvent.


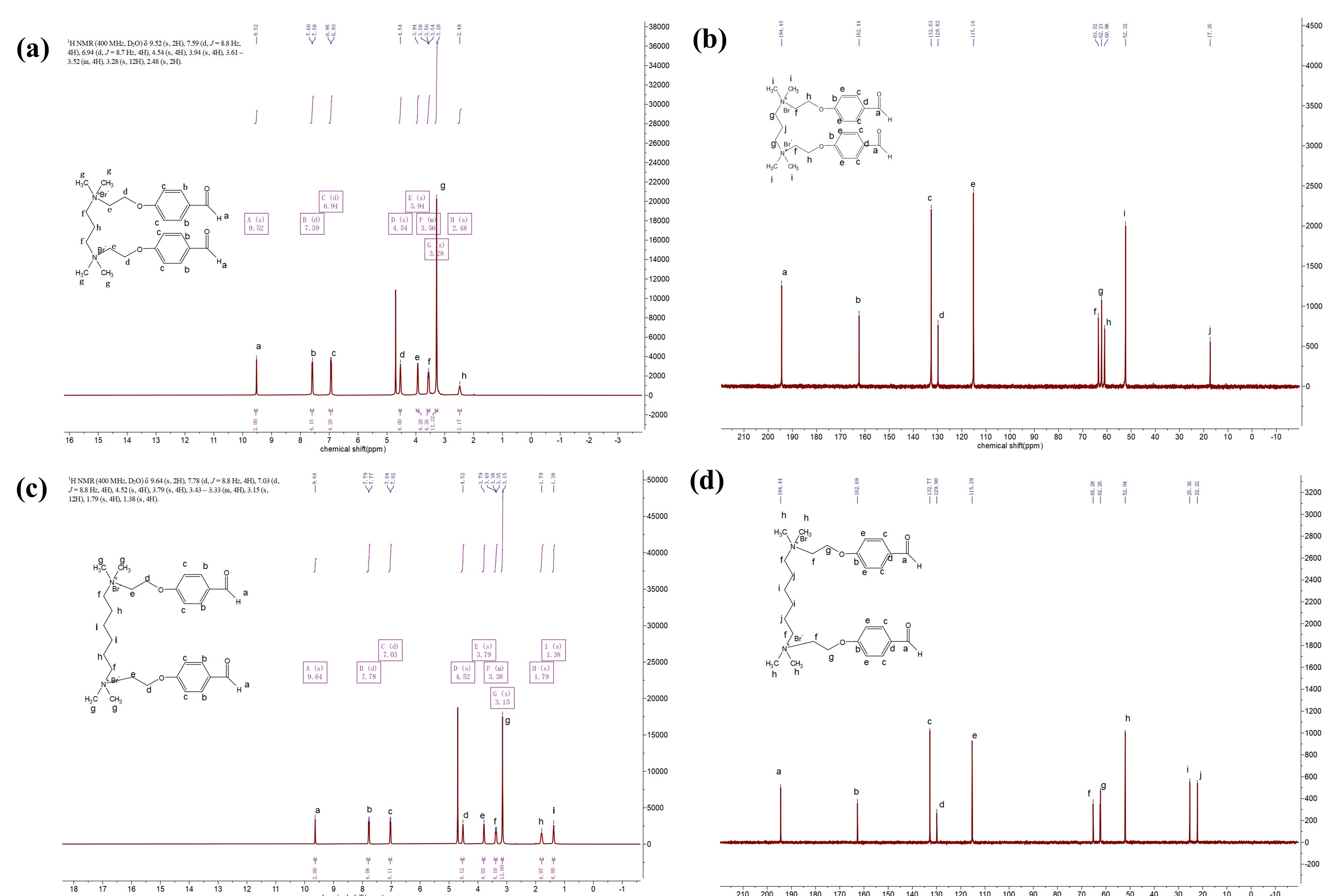
**Fig. S2.** ^1^H NMR spectra (a, c) and ^13^C NMR spectra (b, d) of S_3_ and S_6_ using D_2_O as solvent.


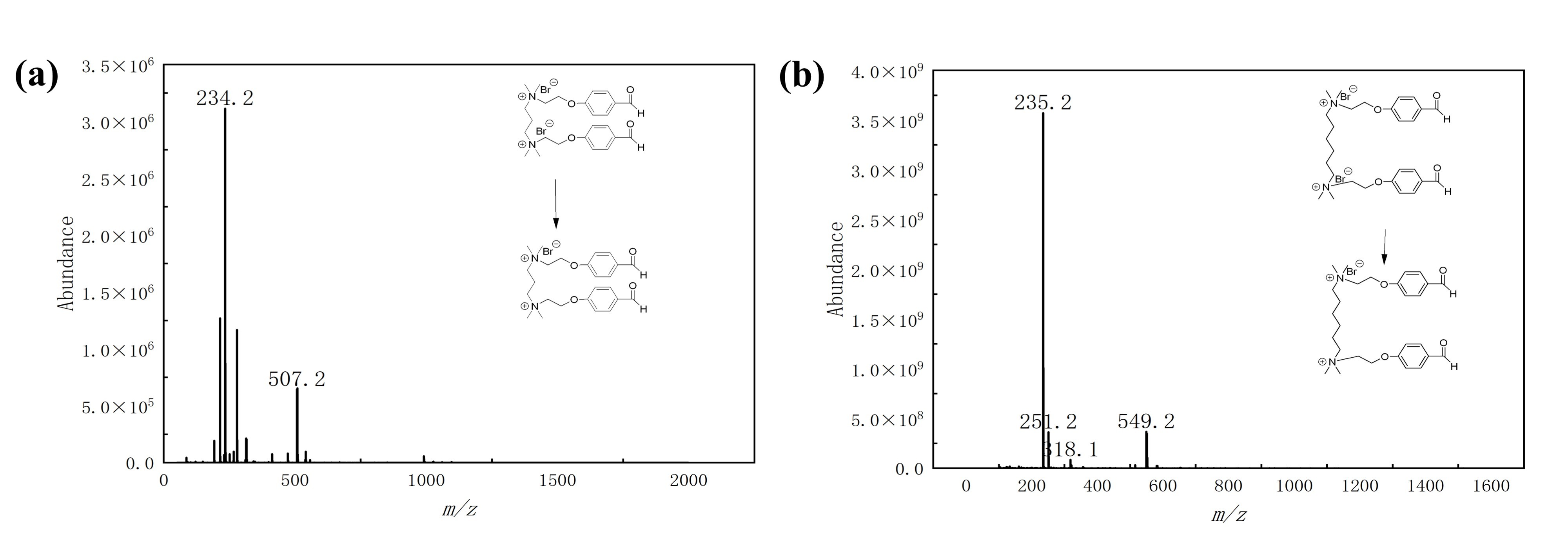
**Fig.S3.** HRMS of S_3_ (a) and S_6_ (b).


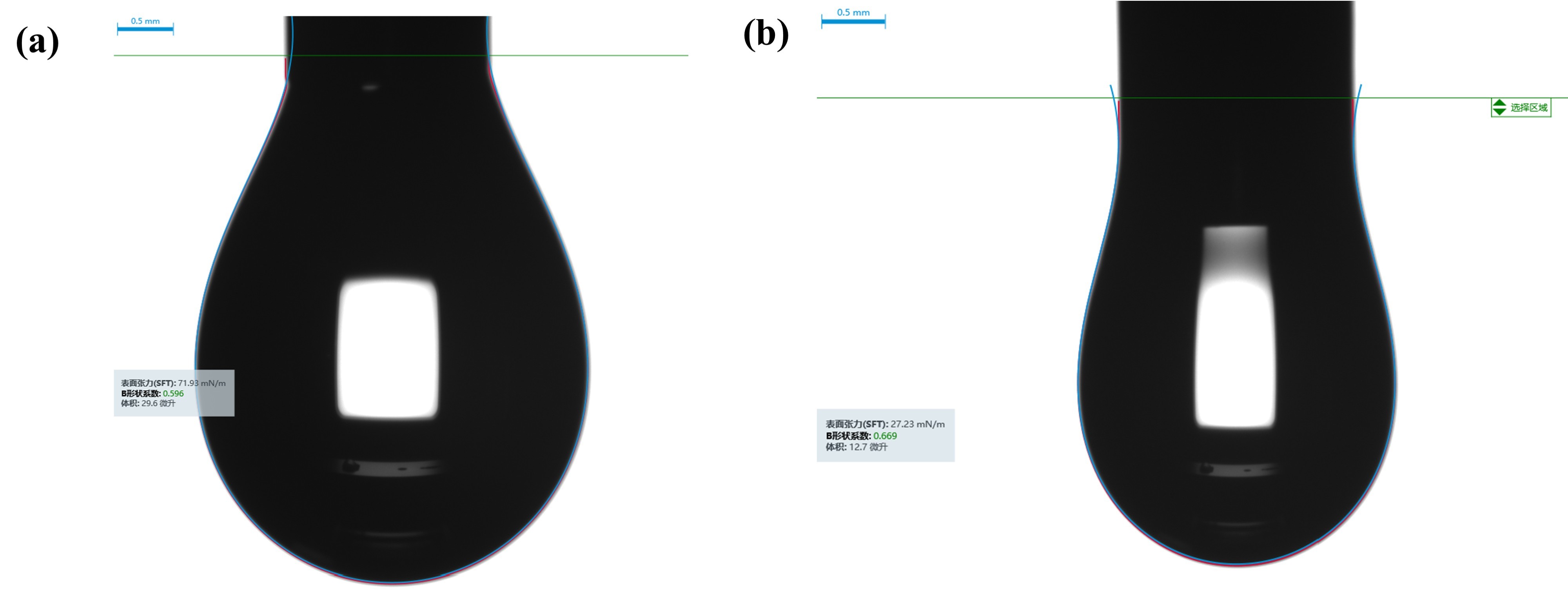


**Fig.S4.** Measured values of surface tension of water(a) and Gemini surfactant(b).


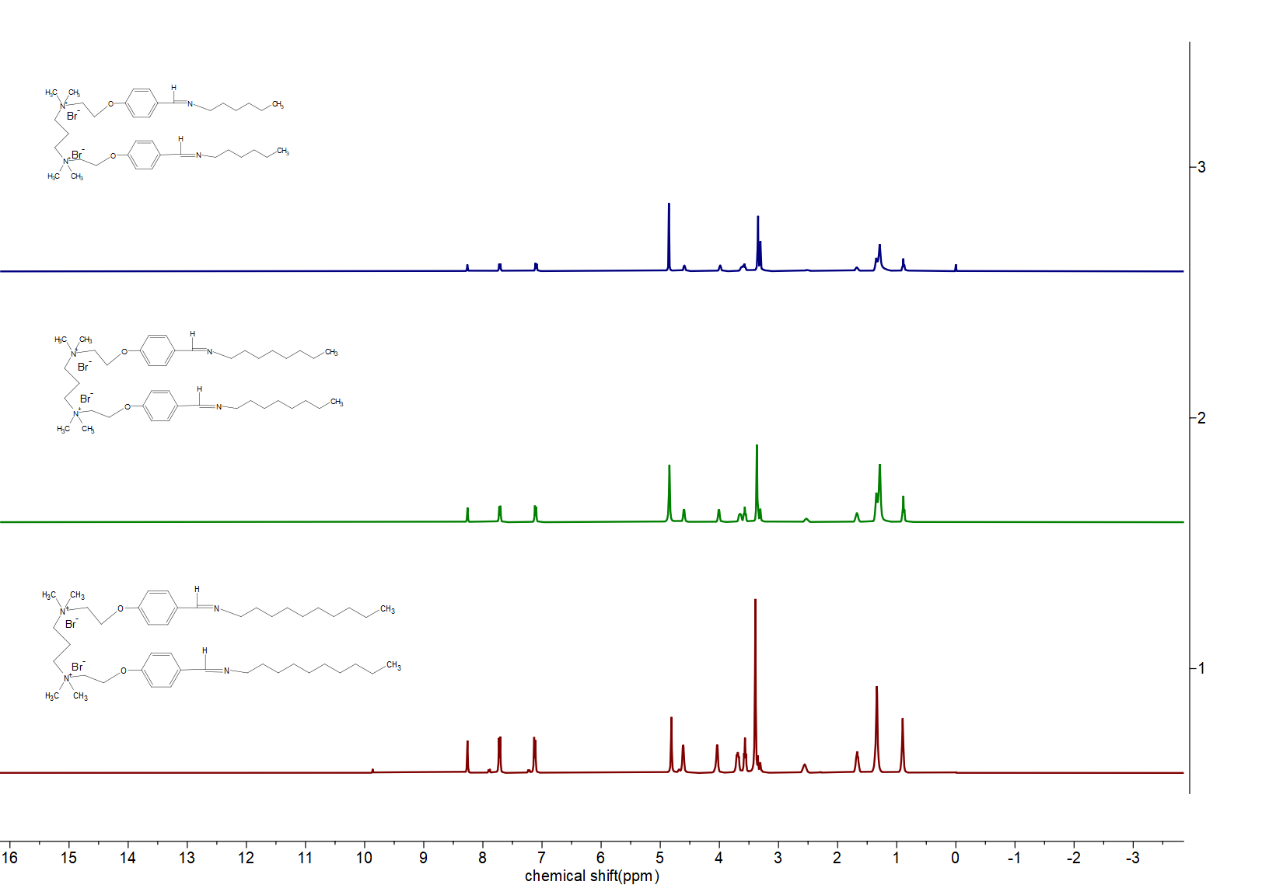


**Fig.S5.**^1^H NMR spectrum of S_3_-T_6_, S_3_-T_8,_ S_3_-T_10_ using CH_3_DO as solvent.


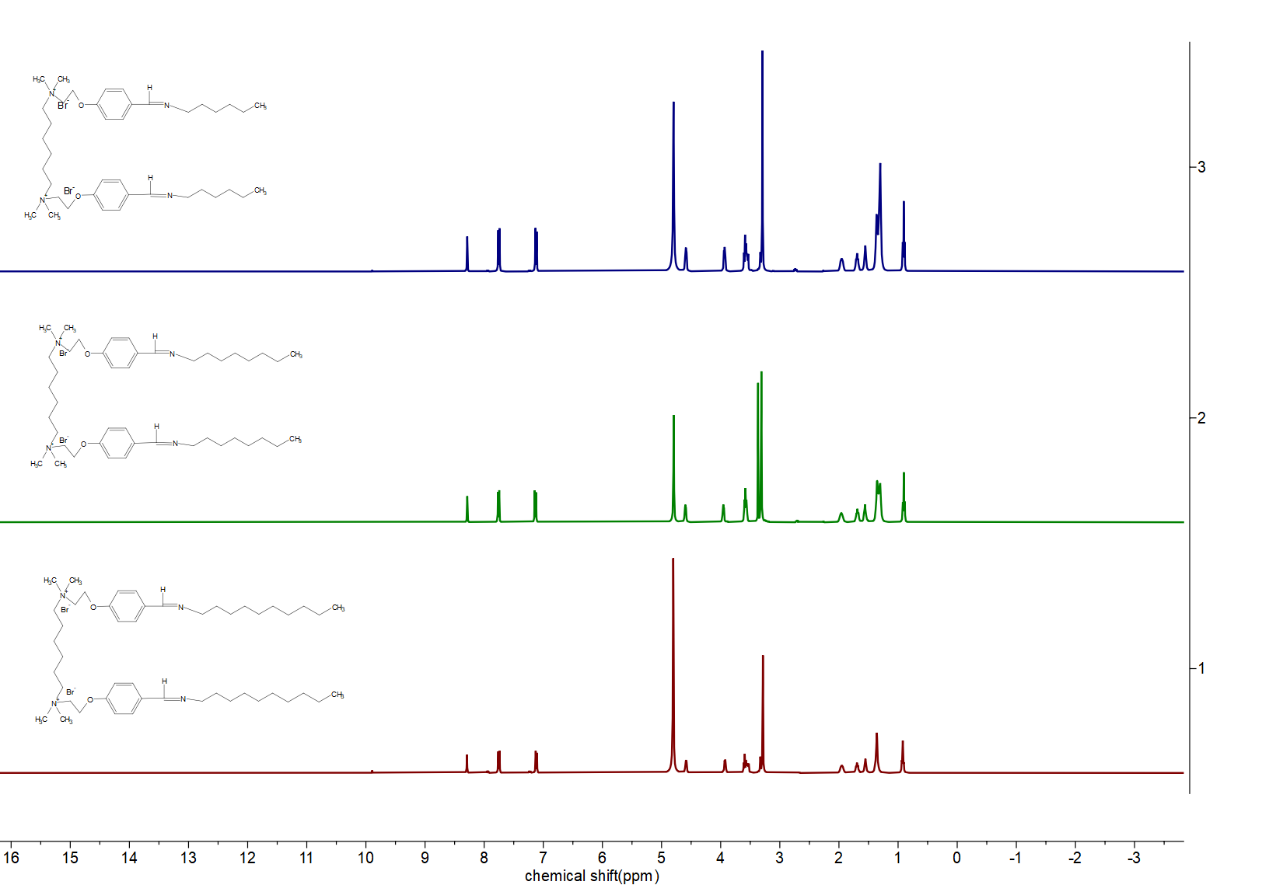


**Fig.S6.** ^1^H NMR spectrum of S_6_-T_6_, S_6_-T_8_, S_6_-T_10_ using CH_3_DO as solvent.


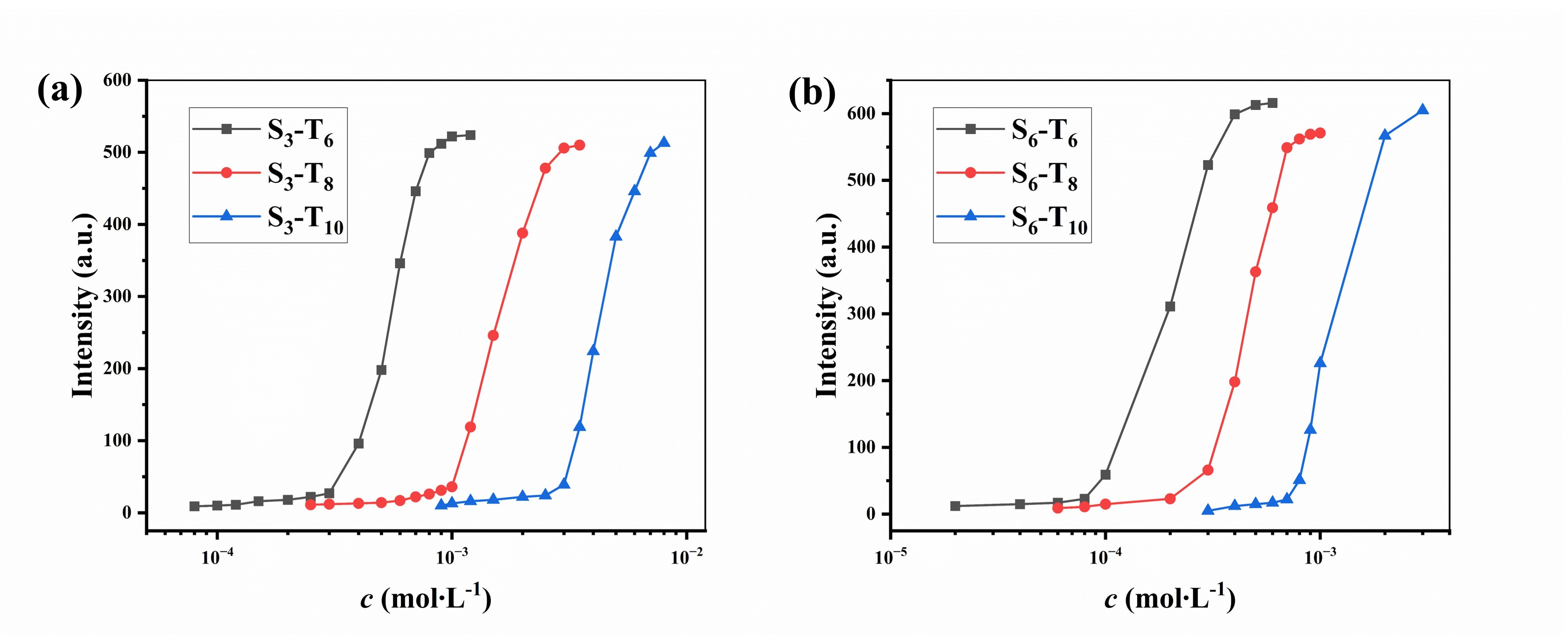


**Fig.S7.** Variations in the intensity of the Nile red fluorescence with concentration of Gemini surfactant based on S_3_ (a) and S_6_ (b) at 25 ℃.


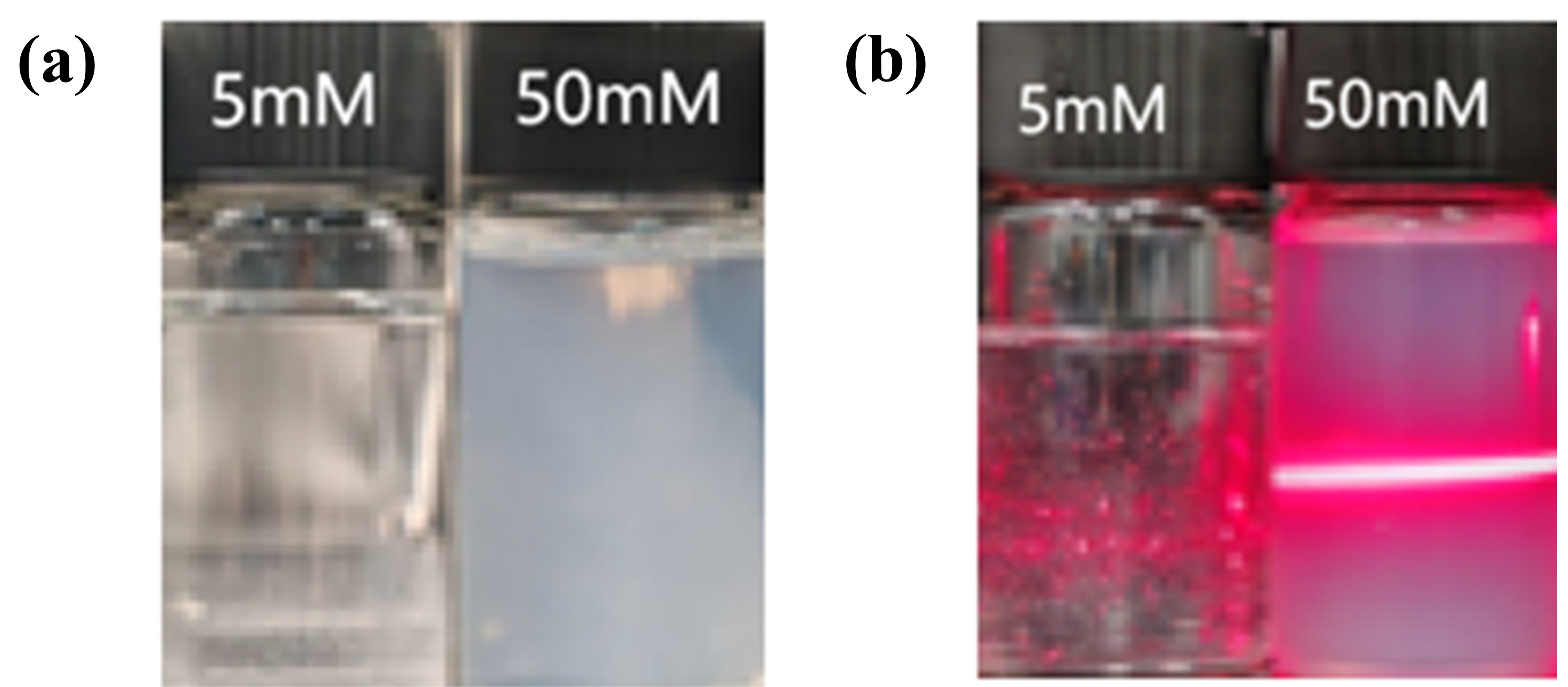


**Fig.S8.** The transparency(a) and tyndall-phenomenon(b) in aqueous solutions of S_6_-T_8_.


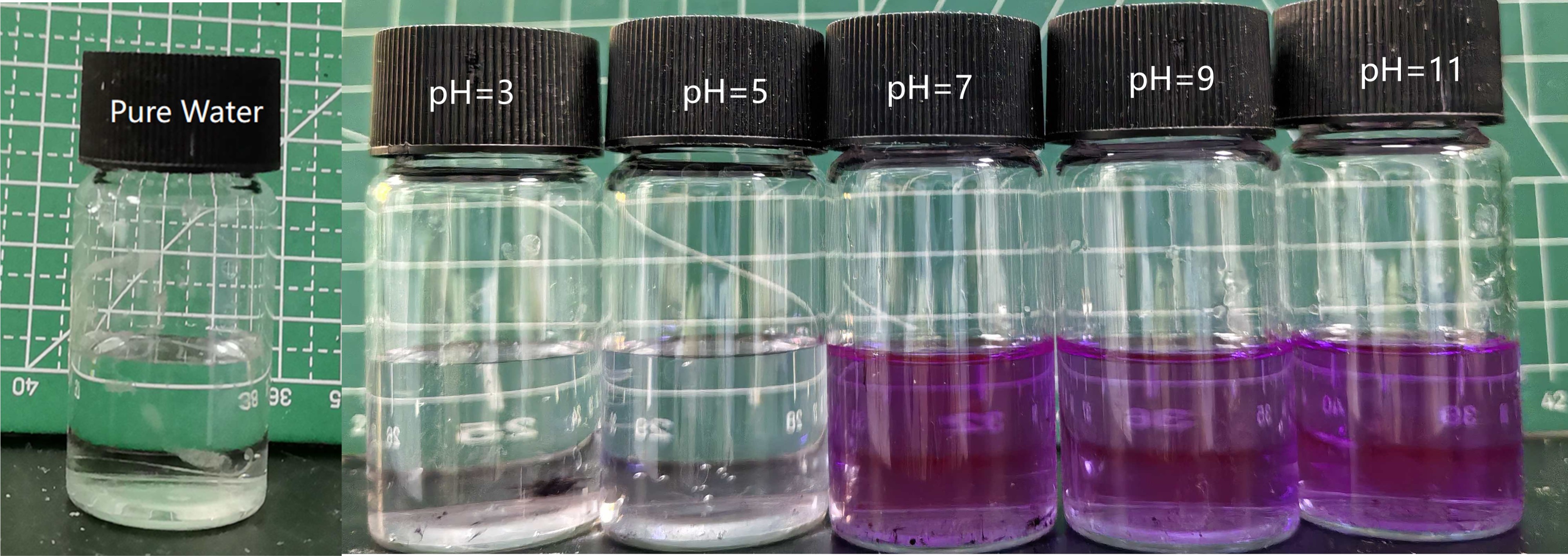


**Fig.S9.** Appearance of mixed solution of surfactant and Nile red at different pHs.


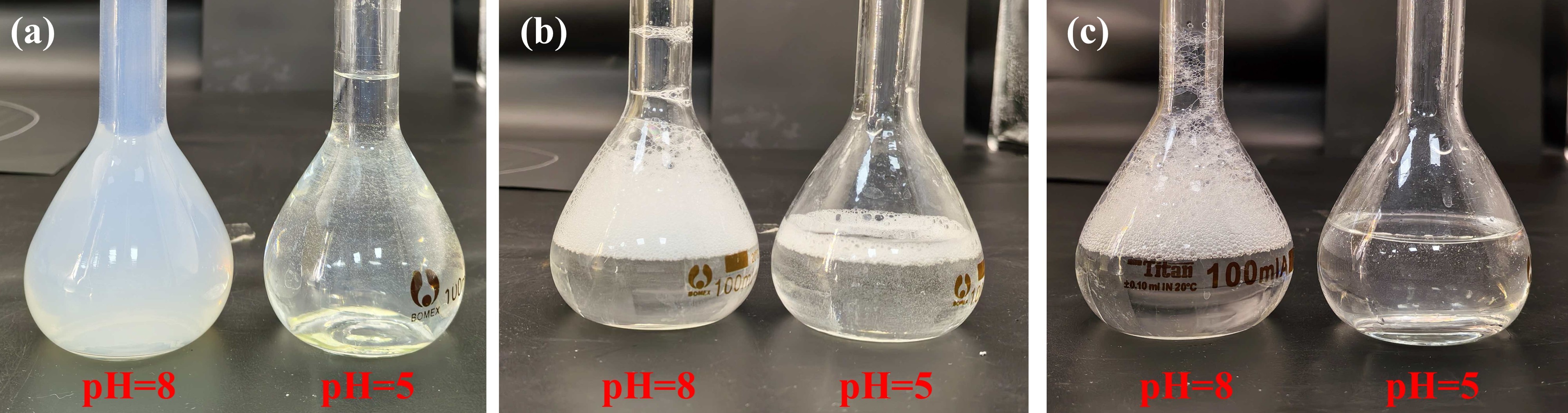


**Fig.S10.** Photos of solutions of S_6_-T_10_(a), oscillated solutions of S_6_-T_8_(b) and standing for a period of time(c).
